# Switchable gate-opening effect in metal–organic polyhedra assemblies through solution processing†
†Electronic supplementary information (ESI) available: Experimental procedures, crystallographic tables, thermogravimetric data, volumetric gas sorption data for N_2_ and CO_2_, additional powder X-ray diffraction data, and *in situ* powder X-ray diffraction-gas sorption experiments (PDF). X-ray crystallographic data in CIF format for **1-DMF**, **1-THF**, and **2-DMA**. CCDC 1840858–1840860. For ESI and crystallographic data in CIF or other electronic format see DOI: 10.1039/c8sc02263a


**DOI:** 10.1039/c8sc02263a

**Published:** 2018-07-10

**Authors:** Gavin A. Craig, Patrick Larpent, Shinpei Kusaka, Ryotaro Matsuda, Susumu Kitagawa, Shuhei Furukawa

**Affiliations:** a Institute for Integrated Cell-Material Science (WPI-iCeMS) , Kyoto University , Yoshida, Sakyo-ku , Kyoto 606-8501 , Japan . Email: shuhei.furukawa@icems.kyoto-u.ac.jp; b Department of Synthetic Chemistry and Biological Chemistry , Graduate School of Engineering , Kyoto University , Katsura, Nishikyo-ku , Kyoto 615-8510 , Japan

## Abstract

Gate-opening gas sorption is known for metal–organic frameworks, and is associated with structural flexibility and advantageous properties for sensing and gas uptake. Here, we show that gate-opening is also possible for metal–organic polyhedra (MOPs), and depends on the molecular organisation in the lattice. Thanks to the solubility of MOPs, several interchangeable solvatomorphs of a lantern-type MOP were synthesised *via* treatment with different solvents. One phase obtained through use of methanol induced a gate-opening effect in the lattice in response to carbon dioxide uptake. The sorption process was thoroughly investigated with *in situ* powder X-ray diffraction and simultaneous adsorption experiments. Meanwhile, solution processing of this flexible phase using THF led to a permanently porous phase without a gate-opening effect. Furthermore, we find that we can change the metallic composition of the MOP, and yet retain flexibility. By showing that gate-opening can be switched on and off depending on the solvent of crystallisation, these findings have implications for the solution-based processing of MOPs.

## Abstract 2

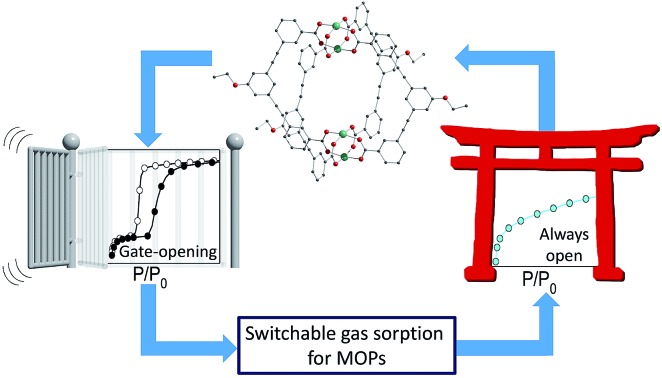
Assemblies of lantern-type metal–organic polyhedra are found to show gate-opening gas adsorption depending on how they are treated with solvents.

## Introduction

Cooperative phenomena are found in many areas of chemistry. For example, cooperative models with a sharp increase in the rate of self-assembly have been proposed for supramolecular polymerisation,^1^ while cooperativity in spin crossover complexes leads to abrupt switches in the spin state of certain transition metals.^2^ Metal–organic frameworks (MOFs), a type of polymeric material consisting of metal nodes bridged by organic ligands, may also show cooperativity.^3^ Usually, this results in a structural transition triggered by the entry of a gas molecule to the lattice, with a sudden increase in sorption capacity of the framework – so-called gate-opening behaviour.^4^ The extended character of MOFs is important here, as it often means that they retain crystallinity after activation, and such long-range order rapidly propagates the structural transformation throughout the lattice.^5^

Metal–organic polyhedra (MOPs) are the porous molecular counterparts of MOFs. This type of porous molecular solid is an attractive class of materials because their discrete nature means that they can be highly soluble, which is advantageous for solution-based processing.^6^ The reticular chemistry used for MOFs can be applied to the design of MOPs, allowing precise control over the shape of the molecule and the size of the pore.^7^ The intrinsic pores of MOPs should ensure that the activated solids display porosity. However, the weak nature of the intermolecular interactions, which leads to high solubility, can often cause the lattice to collapse, resulting in an absence of porosity or slow gas diffusion.^8^ Additionally, the crystal packing of porous molecules is not straightforward to predict, as shown by recent combined theoretical and experimental work on organic cages, which revealed the huge range of potential lattice arrangements for a given porous molecule.^9^ Here we show the effect of chemical modification through covalent and coordination bonds at the molecular periphery on the arrangement of MOPs in the solid state and the discovery of gate-opening behaviour for MOPs. We have observed both type I gas sorption and stepped gate-opening for the same MOP molecule. *In situ* powder X-ray diffraction (PXRD) has been used to follow the gate-opening process, and demonstrates that these phenomena are intimately related to the crystal packing of the activated phase. It is possible to interconvert between these soft and rigid activated phases by treating the samples with the appropriate solvents. We further show that we can change the metallic composition of the MOP but retain the same flexible activated phase that leads to gate-opening gas sorption, at the same time modulating the pressure at which gate-opening occurs.

## Results and discussion

### Design and synthesis of the ligand and MOPs

**Chart 1:**
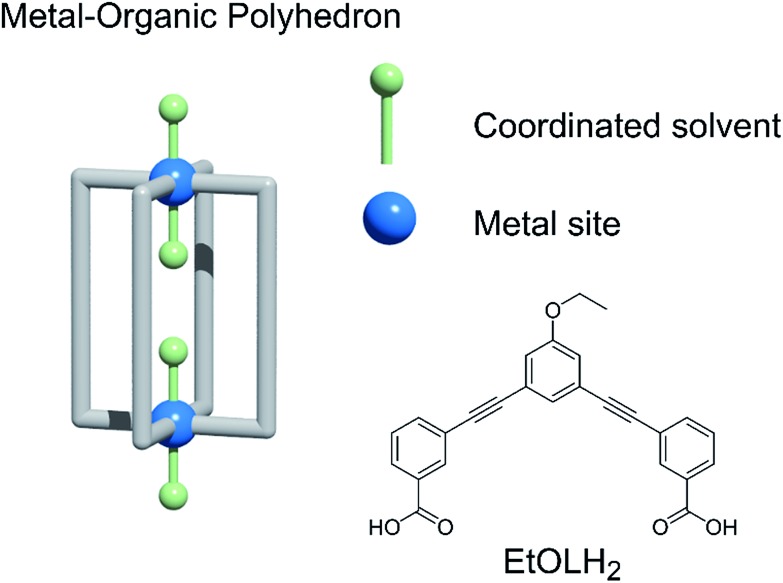
(Left) A blueprint for lantern-MOPs. (Right) The features we vary in the work to modify the gas sorption properties of the MOPs described here, and the ligand used.

The organisation of a MOP in the solid state will depend on its shape, the influence of solvent molecules on the packing, and the combination of intermolecular interactions arising from the functional groups present. The core shape of the MOP is determined by the geometry of the linker, while the packing can be altered by functionalisation of the external backbone of the ligand.^10^ Among potential MOPs for porous solids, we chose a lantern-type MOP with four organic linkers bridging two paddlewheel nodes: this shape guarantees relatively strong molecular interactions through effective pi–pi stacking, in contrast to other conventional Archimedean MOPs such as the cuboctahedron.^11^ MOPs with labile coordination sites at the axial sites of paddlewheel nodes, can be conveniently recrystallised from different solvents, allowing the investigation of the solvent effect on molecular packing arrangement (Chart 1).^12^

**Fig. 1:**
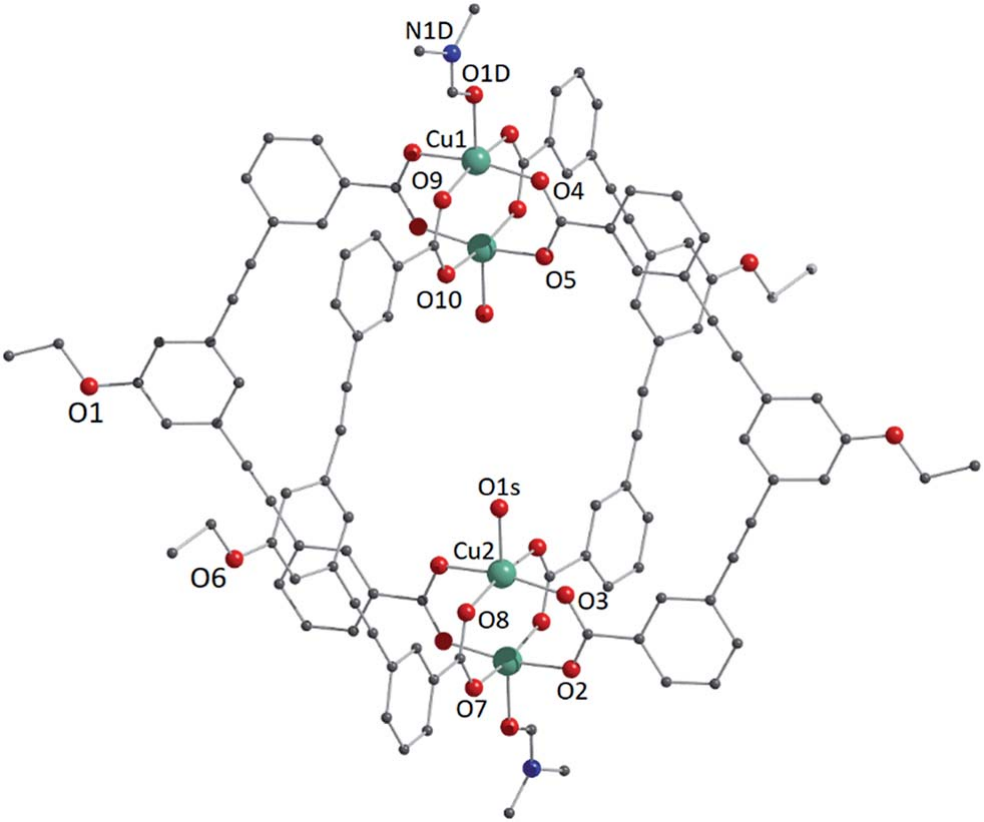
View of the molecular structure of the MOP unit in compound **1-DMF**. Hydrogen atoms have been omitted for clarity, and only crystallographically independent metal ions and heteroatoms have been labelled.

We studied two lantern MOP molecules based on the assembly of two paddlewheel nodes (Cu_2_ for **1**, Rh_2_ for **2**) with the deprotonated form of the ligand 3,3′-((5-ethoxy-1,3-phenylene)bis(ethyne-2,1-diyl))dibenzoic acid (EtOLH_2_ in Chart 1; see the ESI† for full synthetic details). We prepared this ligand as a functionalised version of the ligand 3,3′-((1,3-phenylene)bis(ethyne-2,1-diyl))dibenzoic acid (LH_2_), which has been used previously to synthesise both Cu- and Rh-lantern MOPs.^13^ Compound **1** can be obtained as single crystals *via* reaction of Cu(OAc)_2_·H_2_O with EtOLH_2_ in DMF at room temperature, yielding the neutral complex [Cu_4_(EtOL)_4_(H_2_O)_2_(DMF)_2_] (**1-DMF**), or as a bulk powder by performing the reaction in DMA and forcing precipitation through addition of MeOH. The molecular structure of **1-DMF** is shown in Fig. 1. The compound crystallises in the triclinic space group *P*1[combining macron], with half of the MOP molecule in the asymmetric unit and one entire molecule in the unit cell. The molecule consists of two Cu_2_ paddlewheel nodes arranged on either side of the crystallographic inversion centre and bridged through four EtOL^2–^ ligands. A DMF molecule is bound to the exterior Cu1 of the paddlewheel in the axial position through the oxygen atom. Meanwhile, the coordination sphere of the Cu2 on the interior site is completed by a water molecule. The pore size of this MOP can be defined by the distance across the pore of the molecule between Cu2 ions of 9.308(1) Å and the average distance between the opposing ethoxyphenyl rings of 14.838(6) Å. The crystal packing is dominated by π···π stacking interactions arising from the faces of the aromatic ligand (Fig. S2†). The shortest interaction between aromatic rings on neighbouring MOP molecules measures 3.450(1) Å. These interactions lead to an arrangement displaying ordered channels along the [010] direction of the crystal lattice (*vide infra*). The shortest distance between the centre of two pores in the lattice is 15.965(1) Å. In the crystal lattice of the compound [Cu_4_(L)_4_(DMF)_4_] the shortest distance between the centre of two pores was 11.245(2) Å,13*a* illustrative of a more tightly packed lattice, which may be due to the ethoxy-group in **1-DMF**.

### Gate-opening sorption behaviour in the flexible lattice of a MOP

**Fig. 2:**
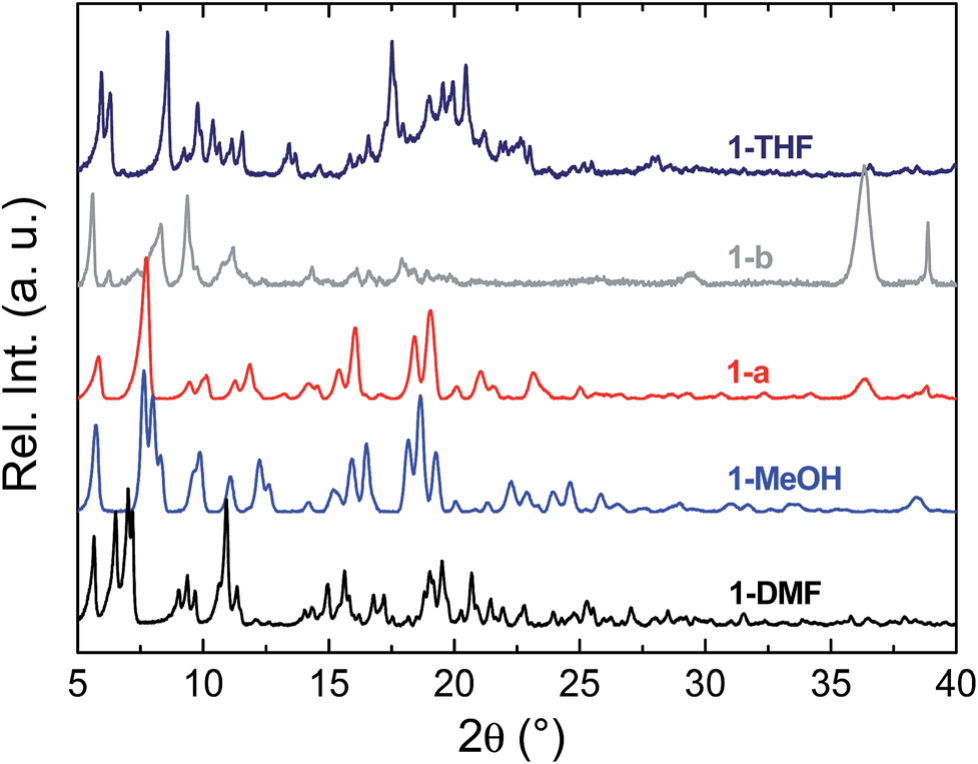
PXRD data collected for the as-synthesised crystals **1-DMF** (black), and subsequent to solvent exchange with MeOH (**1-MeOH**, blue). The data collected after the activation processes for **1-MeOH** (leading to **1-a**, red) and **1-THF** (leading to **1-b**, grey) are shown. The diffractogram for **1-THF** is shown in navy blue.

To prepare compound **1** for activation, the crystals of **1-DMF** or of the bulk polycrystalline powder from DMA were soaked in MeOH for seven days, to exchange out the less volatile DMF or DMA molecules. The blue powder yielded by this solvent exchange process is crystalline; however, PXRD measurements showed that the crystal packing was altered with respect to that found in **1-DMF**, and this new phase is referred to as **1-MeOH** (Fig. 2). To activate **1** for gas sorption measurements, **1-MeOH** was heated overnight at 120 °C under vacuum (Fig. S3 and S4†). The PXRD data collected *in situ* of the resulting activated phase show that under these conditions, **1** packs in a third, distinct phase (**1-a**). These successive transformations demonstrate the importance of the solvent molecules for the overall molecular organisation.

**Fig. 3:**
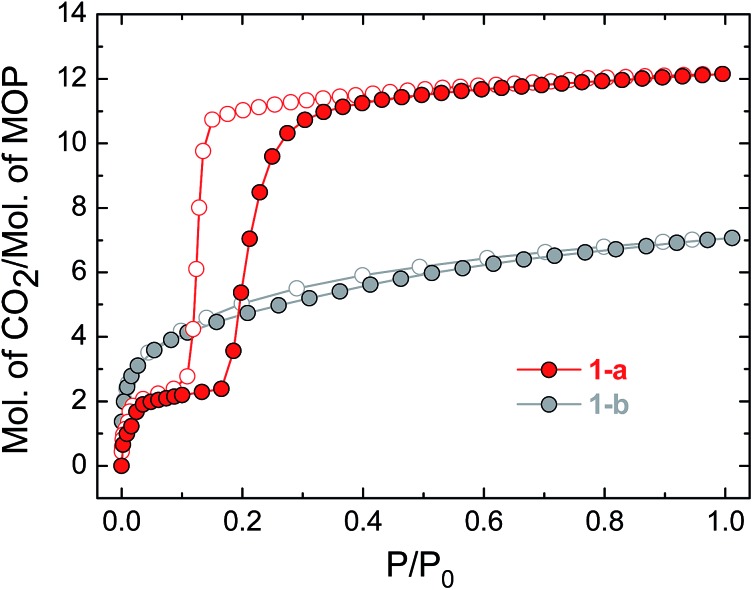
CO_2_ sorption isotherms for compounds **1-a** and **1-b**, measured at 195 K. Filled circles represent adsorption and empty symbols desorption in the colours indicated.

The uptake of N_2_ by **1-a** at 77 K was found to be very low, indicating that this phase is essentially non-porous towards N_2_ (Fig. S5† shows the volumetric N_2_ and CO_2_ sorption isotherms for **1-a**). The gas sorption of CO_2_ by **1-a** at 195 K is radically different (Fig. 3). There is a micro-porous region in the low pressure regime, where *P*/*P*_0_ < 0.035, before the isotherm enters a plateau for two adsorbed molecules of CO_2_ for each molecule of MOP. This plateau persists until a sharp increase in the uptake of CO_2_ is triggered at *P*/*P*_0_ ≈ 0.18. This gate-opening effect is steepest in the pressure range 0.18 < *P*/*P*_0_ < 0.40, before levelling out at higher pressures, attaining a maximum capacity of 12 adsorbed molecules of CO_2_ for each molecule of MOP at *P*/*P*_0_ = 1. The desorption measurements show gate-closing to occur at *P*/*P*_0_ ≈ 0.15, with the isotherm returning to the plateau of two adsorbed molecules of CO_2_ at *P*/*P*_0_ = 0.08. This behaviour is in stark contrast to the CO_2_ sorption isotherm described for the related MOP [Cu_4_(L)_4_], which was found to present a type I isotherm.13*a* This behaviour is also different from the kinetic effect of slow gas diffusion observed for a handful of MOPs in the form of unstructured hysteresis loops in their gas sorption isotherms.^14^

**Fig. 4:**
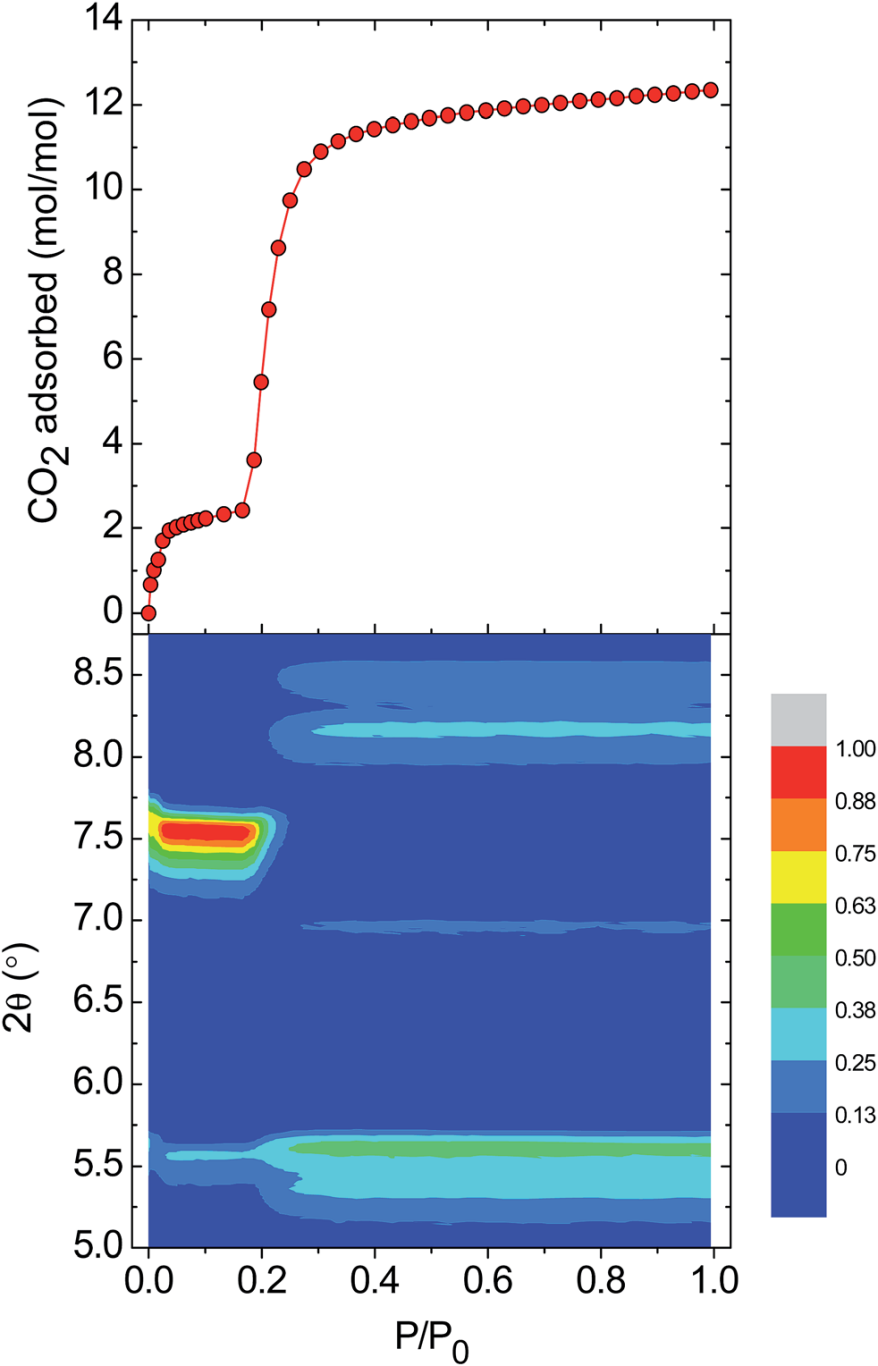
(top) The adsorption branch of the CO_2_ uptake measurements performed at 195 K on **1-a**. (bottom) A contour plot of the evolution of the Bragg peaks over the range 2*θ* = 5.0–8.75° as a function of *P*/*P*_0_. The intensity of the peaks is relative to the strong peak observed at 7.54° over the pressure range *P*/*P*_0_ = 0.035–0.18.

Gate-opening gas sorption is a well-known phenomenon for MOFs, and requires a highly ordered network together with structural transformability. These features lead to the cooperative character in the lattice that allows a collective structural transition to occur. In the case of MOPs, however, the lattice often collapses to an amorphous phase upon activation.8d,^15^ To characterise the changes in crystallinity driven by the entry of CO_2_ into the lattice of **1-a**, we performed *in situ* CO_2_ sorption and PXRD measurements. Fig. 4 shows the evolution of the Bragg peaks in the range 2*θ* = 5–8.75° as *P*/*P*_0_ is increased from 0 to 1 (selected individual diffractograms are given in Fig. S6†). The initial gas uptake in the microporous regime (0.00 < *P*/*P*_0_ < 0.035) induces a displacement of the peak at 2*θ* = 7.62° to 7.54°, accompanied by an increase in intensity. This new phase is retained over the range (0.035 < *P*/*P*_0_ < 0.18), and corresponds to the plateau observed in the gas sorption isotherm for two adsorbed molecules of CO_2_. In fact, the majority of the observed peaks in the PXRD diffractogram are displaced to lower values of 2*θ* for *P*/*P*_0_ < 0.18, which suggests an expansion of the unit cell in this regime. Therefore, it could be suggested that the first event in the lattice is entry of CO_2_ and formation of an interaction with the external Cu(ii) ions of the MOP paddlewheel, causing the expansion of the lattice and the existence of the plateau in the volume of CO_2_ adsorbed, which corresponds to two molecules of gas. At *P*/*P*_0_ ≈ 0.20, a phase transition occurs: the peak at 2*θ* = 7.54° disappears at the same time as the emergence of new peaks at 2*θ* = 8.45, 8.15, 6.95, and 5.35°. The transformation from a closed phase of **1** to an open phase causes the sharp increase in gas uptake observed in the sorption isotherm. The open phase is retained through continued adsorption and through the desorption measurements until lowering the pressure to *P*/*P*_0_ ≈ 0.15 where the gate-closing occurs.

### Switching gas sorption behaviour through solvent treatment

**Fig. 5:**
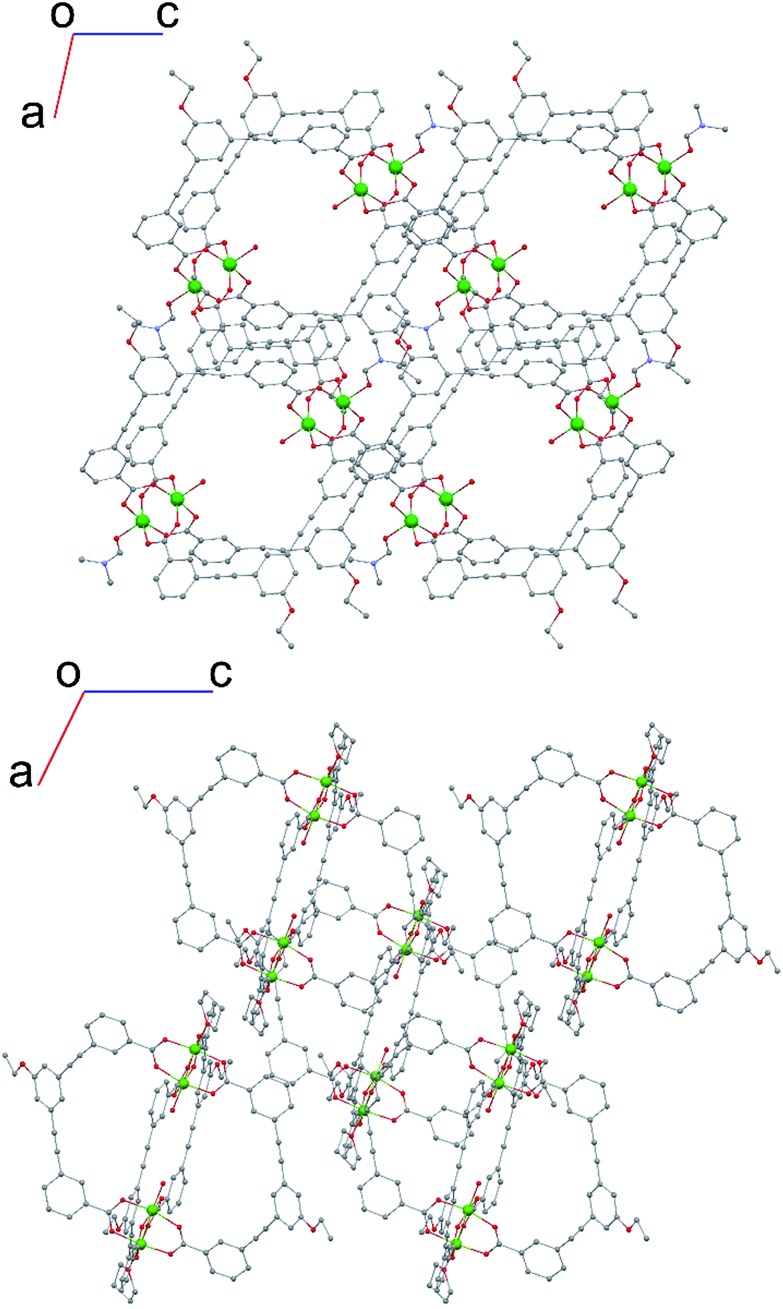
(top) View along the *b*-axis for **1-DMF** and (bottom) view along the *b*-axis for **1-THF**, emphasising the different crystal packing displayed by both solvatomorphs.

**Fig. 6:**
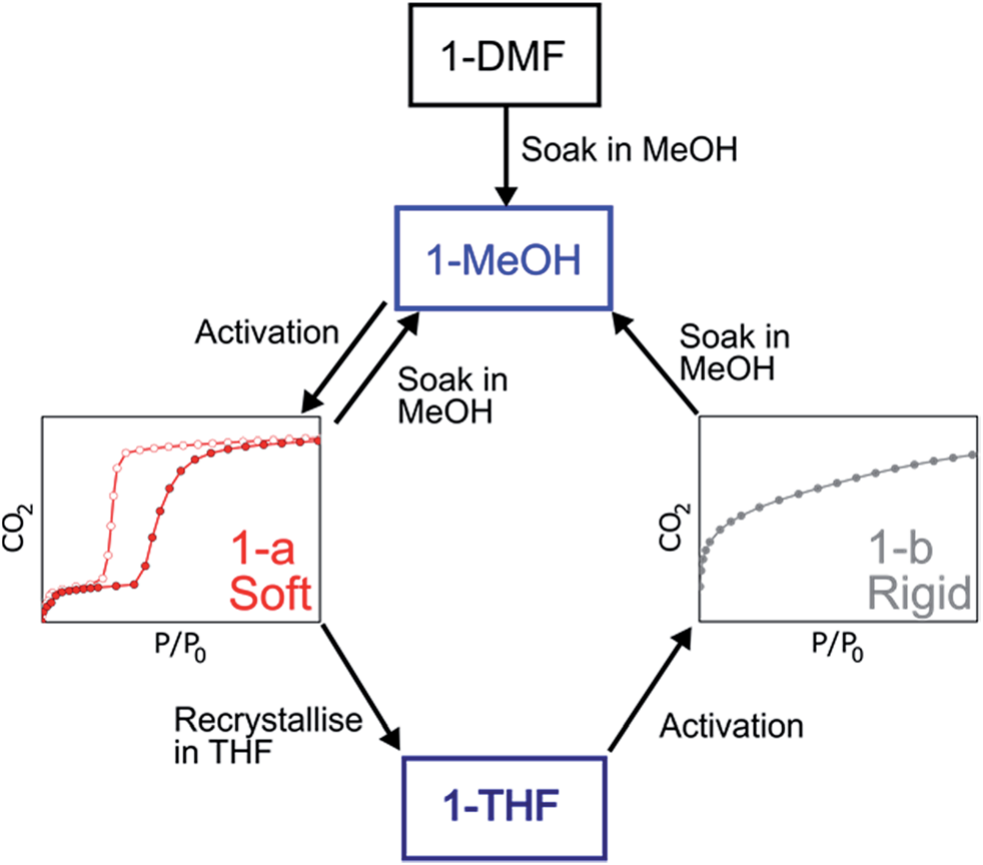
Summary of the transformations described for the MOPs based on **1**.

The structure of the activated phase of a MOF is generally independent of the solvent removed from the pores, due to the infinite nature of the framework. A recent, notable exception was described for an indium-containing MOF SHF-61,^16^ where activation from the CHCl_3_ solvate led to an open activated phase, while activation from the DMF solvate yielded a closed activated phase.^17^ Given the role of solvent molecules in determining the crystal packing of the MOP molecules in compound **1**, we targeted a new crystalline phase by re-crystallising the activated phase **1-a** from THF. The molecular structure of the solvatomorph, **1-THF**, is shown in Fig. S7.† In contrast to the triclinic phase **1-DMF**, **1-THF** packs in the higher symmetry monoclinic space group *P*2_1_/*n*, and the conformation of the MOP molecule is distinct (Fig. S8†). Here, the axial sites of the exterior Cu(ii) ions are occupied by THF molecules, while water molecules coordinate to the Cu(ii) ions of the interior site. The effect of the THF molecules is to induce a completely distinct packing arrangement in **1-THF** with respect to **1-DMF**, in that there are no ordered channels present (Fig. 5). The shortest distance between the centre of the pores in **1-THF** is 16.222(3) Å (*cf.* 15.965(1) Å in **1-DMF**). The phase **1-THF** was activated by drying with supercritical CO_2_ prior to gas sorption experiments. Removal of THF from the lattice of **1-THF** did not lead to the same solvent free phase **1-a** that was obtained from the activation of **1-MeOH**. Instead, a new phase **1-b** was obtained, which had a lower degree of crystallinity than **1-a** (Fig. 2). The consequences of this alternative packing arrangement for the gas adsorption properties of **1** are shown in Fig. 3, where a type I isotherm is observed for the uptake of CO_2_ at 195 K, with a lower overall capacity. As with **1-a**, there is negligible uptake of N_2_ at 77 K (Fig. S9†). For both **1-a** and **1-b**, it is likely that initial CO_2_ uptake occurs with adsorption to the open metal sites (OMS) of some external Cu(ii) sites, causing an expansion of the lattice that is sufficient in the case of **1-b** to allow continued filling of the pores. For **1-a**, the stepped nature of the isotherm suggests a more ordered process, in which all of the external OMSs are coordinated by CO_2_ before incorporation of CO_2_ into other sites of the lattice. Whichever sorption process is active depends on the initial packing of the activated phase, which in turn is determined by the solvatomorph used to generate the activated phase. It is possible to reversibly cycle between these solvated phases: by soaking **1-b** in MeOH, the phase **1-MeOH** is recovered, from which **1-a** and **1-b** can be obtained (Fig. 6).

### Influence of the metal nodes of the MOP on the gate-opening effect

**Fig. 7:**
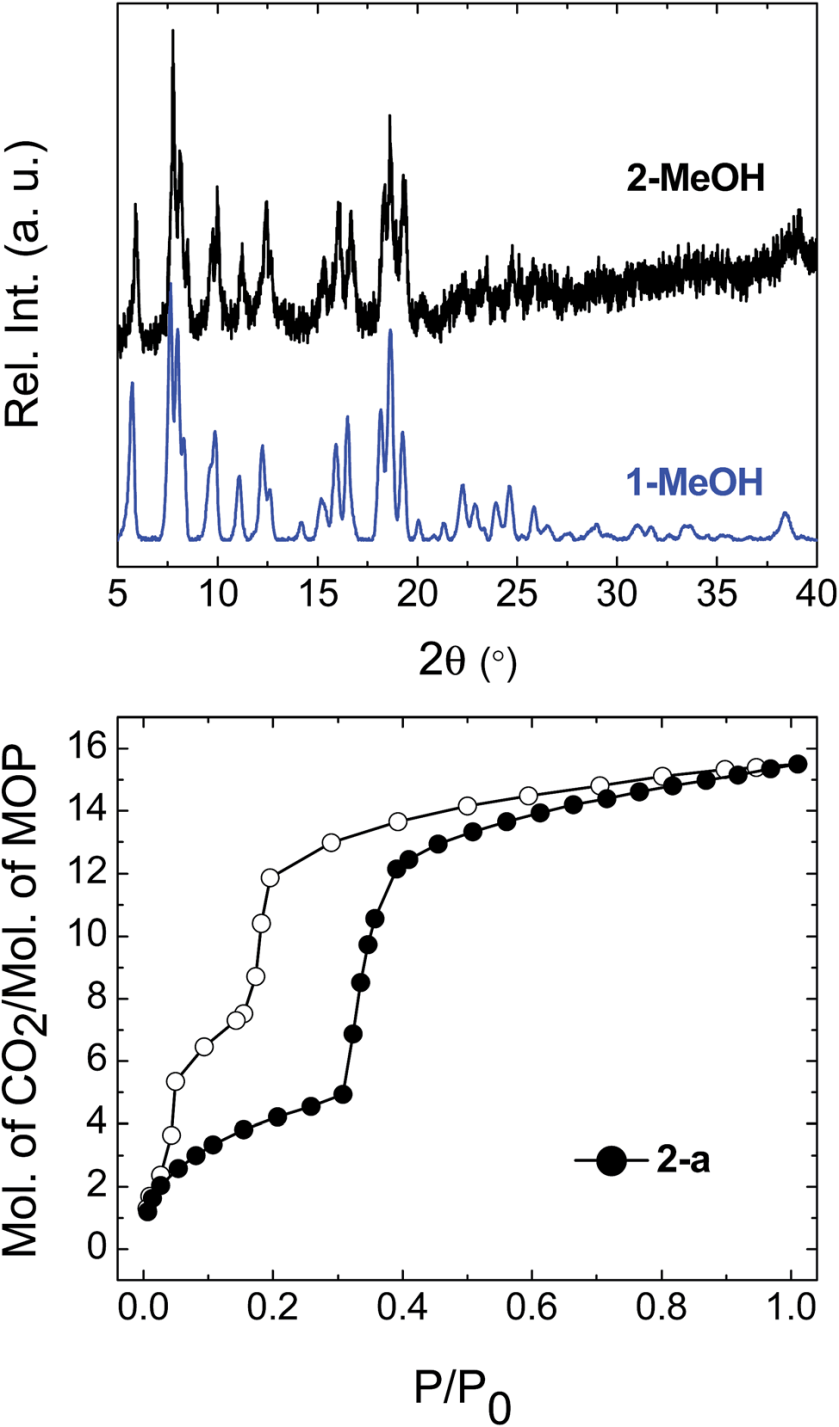
(top) Comparison of the PXRD diffractograms for the flexible phase **1-MeOH** and the phase obtained for the Rh-containing MOP **2-MeOH**. (bottom) CO_2_ sorption isotherms for **2-a**, measured at 195 K. Filled circles represent adsorption and empty symbols desorption.

Having demonstrated that the gas sorption of a specific MOP geometry could be modified by the solvent molecules in the parent solvatomorph, we were interested in the effect of changing the identity of the metal paddlewheel. Thus, a lantern-type MOP was synthesised using [Rh_2_(OAc)_4_] as a starting material, yielding [Rh_4_(EtOL)_4_(MeOH)_4_] [Rh_4_(EtOL)_4_(H_2_O)_2_(DMA)_2_] (**2-DMA**). The as-synthesised complex crystallises in the triclinic space group *P*1[combining macron], with two distinct MOP molecules in the lattice: one with the axial positions of the paddlewheel occupied only by MeOH molecules, and another with the interior sites occupied by water molecules and the exterior sites by DMA. The molecular structure of one of the MOPs in the lattice is shown in Fig. S10.† Through the solvent exchange process outlined for compound **1**, it was possible to obtain the phase **2-MeOH**, which showed the same crystal packing as the Cu(ii) containing analogue **1-MeOH** (Fig. 7). Activation of the parent phase **2-MeOH** similarly led to the formation of the phase **2-a**, confirming the role played by the relationship between the MOP geometry and the crystal packing of the parent phase in determining the molecular arrangement of the resulting activated phase. Fig. 7 shows the CO_2_ sorption isotherm measured at 195 K for **2-a**. The soft porous character of this lattice is seen to be retained despite the substitution of the metal paddlewheel, and again gate-opening is triggered by the uptake of CO_2_. The gate-opening sorption displayed by **2-a** differs from that of **1-a**, in that it presents a stepped hysteresis loop with a less well defined plateau at lower pressures, and a step is observed in the gate-closing with a higher adsorbed volume of CO_2_. Additionally, **2-a** shows a greater overall uptake of gas (Fig. S11†). The *in situ* PXRD experiments showed that while the packing in **2-a** is the same as in **1-a**, the crystallinity was lower, which may lead to a less ordered process with a more gradual transition both structurally and in the uptake of CO_2_ (Fig. S12†). It may also be that the change in the nature of the OMS, caused by the presence of a different metal centre, leads to a different sorption process overall.^18^

## Conclusions

In summary, we have described gate-opening gas adsorption for metal–organic polyhedra, which we were able to observe due to a dependence of the lattice packing on the solvent from which the molecules were crystallised. Clearly, there are implications for solution-based processing and applications, where different gas uptake would arise from the same molecule depending on how it was crystallised. The fact that cooperative gas uptake depends on how the sample is prepared could also mean that this phenomenon is widespread among metal–organic polyhedra, and may be revealed across a range of compounds with study of solvent exchange conditions. Further, perhaps by functionalising the ligand backbone or introducing hydrogen bonding motifs it would be possible to increase crystallinity or cooperativity in the activated phases, allowing the nature of the gate-opening to be tuned, analogous to the strategies used to increase cooperativity in spin crossover complexes.^19^ The subtlety of the relationship between the different packing motifs also suggests the applicability of recently described energy–structure–function maps to target specific properties in these molecules.^20^

## Experimental

### Synthesis

The full synthetic route for preparation of the ligand EtOLH_2_ is given in the ESI.†

#### 3,3′-((5-Ethoxy-1,3-phenylene)bis(ethyne-2,1-diyl))dibenzoic acid (EtOLH_2_)

Diethyl 3,3′-((5-ethoxy-1,3-phenylene)bis(ethyne-2,1-diyl))dibenzoate (0.850 g, 1.82 mmol) was dissolved in THF/MeOH (2 : 1, 60 mL), and 10 mL of a 2 M aqueous solution of KOH was added. The mixture was stirred at 55 °C for five hours. The organic solvents were removed *in vacuo*, and the remaining basic solution was acidified to pH = 1 with HCl (6 M), yielding a white suspension. This was filtered, and the white powder washed with water (3 × 100 mL), and then dried under vacuum at 50 °C. Yield 0.650 g, 87%. Elemental analysis (%) found (calc.): C 75.94 (76.09), H 4.59 (4.42). ^1^H-NMR (DMSO-d_6_, 500 MHz, 25 °C) *δ* (ppm): 13.26 (br, 2H), 8.10 (s, 2H), 7.99 (d, 2H, ^3^*J* = 7.7 Hz), 7.81 (d, 2H, ^3^*J* = 7.6 Hz), 7.59 (t, 2H, ^3^*J* = 7.7 Hz), 7.40 (s, 1H), 7.21 (s, 2H), 4.13 (q, 2H, ^3^*J* = 6.7 Hz), 1.35 (t, 3H, ^3^*J* = 6.8 Hz). ^13^C-NMR (DMSO-d_6_, 125 MHz, 25 °C) *δ* (ppm): 166.5, 158.6, 135.4, 132.1, 131.4, 129.6, 129.2, 126.7, 123.6, 122.3, 118.0, 89.0, 88.8, 63.7, 14.5.

#### [Cu_4_(EtOL)_4_(H_2_O)_2_(DMF)_2_] (**1-DMF**)

A solution of EtOLH_2_ (40 mg, 0.1 mmol) in DMF (2 mL) was added to a solution of Cu(OAc)_2_·H_2_O (18.2 mg, 0.1 mmol) in DMF (2 mL). The resulting blue solution was left to stand at room temperature. Blue block crystals suitable for single crystal X-ray diffraction formed after several hours. Yield 49.0 mg, 62%. A bulk sample was also synthesised from DMA as follows: a solution of EtOLH_2_ (200 mg, 0.50 mmol) in DMA (4 mL) was added to a solution of Cu(OAc)_2_·H_2_O (97 mg, 0.5 mmol) in DMA (4 mL). The resulting blue solution was stirred at room temperature for 1 hour before MeOH (8 mL) was added. Over the course of several hours, a blue crystalline powder formed that was isolated by filtration, washed with DMA/MeOH (1 : 1, 2 × 5 mL), and dried in air, yielding 331 mg of a blue powder.

#### **1**-**MeOH**

**1-DMF** or the bulk sample from DMA was transformed into the corresponding methanolic phase by soaking the samples in MeOH for seven days, replacing the MeOH each day with fresh solvent. Yield (starting from 49.0 mg of fresh **1-DMF**) 27.6 mg, 92%. Elemental analysis for **1**·(MeOH)_2_(H_2_O)_2_ (%) found (calc.): C 64.31 (64.04), H 3.66 (3.85).

#### [Cu_4_(EtOL)_4_(H_2_O)_2_(THF)_2_]·10THF (**1-THF**)

The activated sample **1-a** was recrystallised from hot THF (approximately 4 mg mL^–1^ THF). Yield (starting from 6 mg of **1-a**) 3.9 mg, 34%. Upon cooling, blue block crystals suitable for single crystal X-ray diffraction formed.

#### [Rh_4_(EtOL)_4_(H_2_O)_2_(DMA)_2_][Rh_4_(EtOL)_4_(MeOH)_4_] (**2-DMA**)

EtOLH_2_ (198 mg, 0.48 mmol), [Rh_2_(OAc)_4_(MeOH)_2_] (100 mg, 0.20 mmol), and Na_2_CO_3_ (53 mg, 0.50 mmol) were suspended in DMA (7 mL), sealed in a vial, and heated at 100 °C for 72 hours. After cooling to RT, the Na_2_CO_3_ was separated from the resulting green suspension by decantation, and the green suspension was centrifuged. Single crystals of **2-DMA** were obtained after one week by layering a 1 mL aliquot of the supernatant with MeOH (1 : 1, v/v). MeOH (30 mL) was added to the remaining supernatant, and the solid isolated by centrifugation. This green solid was washed with DMA, and then left to stand in MeOH for one week, replacing the MeOH each day, yielding the phase **2-MeOH** (55 mg, 26%, based on Rh). Elemental analysis for **2**·(MeOH)_2_(H_2_O)_4_ (%) found (calc.): C 58.24 (58.36), H 3.47 (3.70).

### Physical characterisation

^1^H and ^13^C NMR spectra were measured with a Bruker Ultrashield 500 plus (500 MHz) spectrometer at 25 °C. Infra-red spectra were collected on neat samples using a Jasco FT/IR-6100 spectrometer. Thermogravimetric analyses (TGA) were performed with a Rigaku model Thermo plus EVO under an atmosphere of N_2_, and using a heating rate of 5 °C min^–1^. Powder X-ray diffraction data were collected at room temperature using a Rigaku SmartLab diffractometer equipped with Cu Kα radiation (*λ* = 1.54056 Å). Gas sorption isotherms were measured at 77 K (N_2_) and 195 K (CO_2_) using a BELSORP-max volumetric adsorption instrument from BEL Japan, Inc. The samples of **1-MeOH** and **2-MeOH** were activated by heating for 16 hours under vacuum at a temperature of 120 °C, while **1-THF** was activated through treatment with super-critical CO_2_, carried out on an SCLEAD-2BD autoclave (KISCO) at 14 MPa and 40 °C for 2 hours, before heating to 120 °C under vacuum for 3 hours to complete activation.

#### *In situ* gas adsorption-PXRD

*In situ* gas adsorption-PXRD measurements for **1-a** and **2-a** were performed on a Rigaku SmartLab diffractometer with Cu Kα radiation connected to a BELSORP-18PLUS volumetric adsorption instrument (BEL Japan, Inc.). The instruments were automated and synchronised with each other, and an X-ray diffraction pattern was obtained at each equilibrium point in the adsorption isotherm.

#### Single crystal X-ray diffraction

All crystallographic intensity data were collected using a Rigaku model XtaLAB P200 diffractometer equipped with a Dectris model PILATUS 200K detector and confocal monochromated Mo Kα radiation (*λ* = 0.71075 Å). All structures were solved within Olex2 (ref. 21) with the ShelXT structure solution program using intrinsic phasing, and refined with ShelXL using least squares minimisation (Table S1†).^22^ For all three structures, the SQUEEZE^23^ algorithm in PLATON^24^ was used to account for areas of diffuse solvent. More detail is provided in the ESI.†

## Conflicts of interest

There are no conflicts to declare.

## Supplementary Material

Supplementary information

Crystal structure data
