# Supplementary material for: Switchable gate-opening effect in metal–organic polyhedra assemblies through solution processing
Source: Chem Sci. 2018 Jul 10;9(31):6463–9. doi: 10.1039/c8sc02263a (PMC6115636; doi:10.1039/c8sc02263a)
Supplement: Supplementary file 1 [file SC-009-C8SC02263A-s001.pdf]

Electronic Supplementary Material (ESI) for Chemical Science.
This journal is © The Royal Society of Chemistry 2018

Supporting Information.

Switchable gate-opening effect in metal-organic polyhedra assemblies through solution processing

Gavin A. Craig,[a] Patrick Larpent,[a] Shinpei Kusaka,[a,†] Ryotaro Matsuda,[a,†] Susumu Kitagawa,[a] and Shuhei Furukawa*[a,b]

[a]Institute for Integrated Cell-Material Science (WPI-iCeMS), Kyoto University, Yoshida, Sakyo-ku, Kyoto 606-8501, Japan

[b]Department of Synthetic Chemistry and Biological Chemistry, Graduate School of Engineering, Kyoto University, Katsura, Nishikyo-ku, Kyoto 615-8510, Japan

Table of Contents

Experimental section: synthesis S3

Experimental section: physical characterization S5

Fig. S2. View of the π···π stacking interactions in the lattice of **1-DMF** S8

Fig S3. Thermogravimetric analysis data for the activated phase **1-a** S8

Fig. S4. ^{1}H-NMR spectrum of the acid digested activated phase **1-a** S9

Fig. S5. Volumetric gas adsorption isotherms for **1-a** S9

Fig. S6. *In situ* PXRD data collected during CO_2 gas sorption experiments for **1-a** S10

Fig. S7. View of the molecular structure of the MOP molecule in **1-THF** S11

Fig. S8. Overlay of the MOP molecule **1** from the phases **1-DMF** and **1-THF** S11

Fig. S9. Volumetric gas adsorption isotherms for **1-b** S12

Fig. S10. View of the molecular structure of one of the MOP molecules in **2-DMA** S12

Fig. S11. Volumetric gas adsorption isotherms for **2-a**. S13

Fig. S12. *In situ* PXRD data collected during CO_2 gas sorption experiments for **2-a** S14

Experimental details.

Synthesis

All reagents were used as purchased from Sigma Aldrich or Wako (Japan).

(i) 3,3′-((5-ethoxy-1,3-phenylene)bis(ethyne-2,1-diyl))dibenzoic acid.

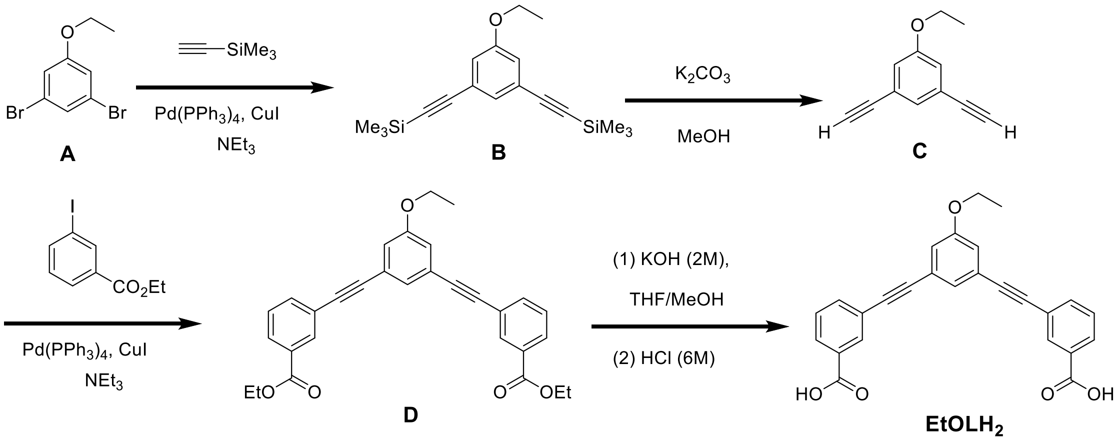

Scheme S1. Synthetic route used to synthesise the ligand 3,3′-((5-ethoxy-1,3-phenylene)bis(ethyne-2,1-diyl))dibenzoic acid.

1,3-Dibromo-5-ethoxybenzene (A). 3,5-dibromophenol (3.0 g, 11.9 mmol), and ethyl p-toluenesulfonate (13 mmol, 1.1 eq) were dissolved in EtOH (150 mL) and degassed with Ar for 10 minutes. K_2CO_3 (3.3 g, 24 mmol) was added, and the resulting mixture was refluxed for 6 hours. After cooling, the resulting suspension was filtered, and the filtrate was evaporated to dryness. The residue was dissolved in CH_2Cl_2 (150 mL) and washed with sat. NH_4Cl (3 × 150 mL) and H_2O (1 × 150 mL). The organic phase was dried over $MgSO_4$, and then evaporated, and the resulting oil purified by column chromatography over SiO_2 with CH_2Cl_2 as eluent, yielding a colourless oil (2.800 g). ^{1}H-NMR ($CDCl_3$, 500 MHz, 25 °C) δ (ppm): 7.20 (s, 1H), 6.96 (s, 2H), 3.99 (q, 2H, ^3J = 7.0 Hz), 1.38 (t, 3H, ^3J = 7.0 Hz). ^{13}C-NMR ($CDCl_3$, 125 MHz, 25 °C) δ (ppm): 160.3, 126.4, 123.2, 117.1, 64.3, 14.7.

((5-ethoxy-1,3-phenylene)bis(ethyne-2,1-diyl))bis(trimethylsilane) (B). 1,3-Dibromo-5-ethoxybenzene (4.900 g, 17.6 mmol) was dissolved in triethylamine (60 mL) and purged with argon. Tetrakis(triphenylphosphine)palladium(0) (0.420 g, 0.36 mmol) and copper (I) iodide (0.100 g, 0.53 mmol) were added under argon flow, and the mixture was cooled to 0 °C. Ethynyltrimethylsilane (5.45 mL, 38.5 mmol) was added dropwise and the orange suspension was heated gradually to 75 °C, and left overnight. The resulting brown slurry was filtered through Celite, and the triethylamine removed *in vacuo*. The orange paste was dissolved with dichloromethane (100 mL) and washed with sat. NH_4Cl (3 × 80 mL) and H_2O (3 × 80 mL). The extracted organic phase was dried over $MgSO_4$, filtered, and reduced to dryness on the rotary evaporator. The resulting brown oil was purified with column chromatography over silica with dichloromethane was eluent, yielding a yellow oil that crystallised to give a pale yellow solid (5.241 g). ^{1}H-NMR ($CDCl_3$, 500 MHz, 25 °C) δ (ppm): 7.18 (s, 1H), 6.92 (s, 2H), 4.00 (q, 2H, ^3J = 6.7 Hz), 1.39 (t, 3H, ^3J = 6.7 Hz), 0.24 (s, 18H).

Ethoxy-3,5-diethynylbenzene (C). ((5-ethoxy-1,3-phenylene)bis(ethyne-2,1-diyl))bis(trimethylsilane) (5.240 g, 16.7 mmol) was dissolved in MeOH (150 mL) and K_2CO_3 (6.500 g, 47.0 mmol) added. The resulting suspension was stirred vigorously at room temperature for four hours, before being filtered and reduced to dryness on the rotary evaporator at a temperature of 30 °C. The resulting yellow paste was dissolved in CH_2Cl_2 and washed with H_2O (3 × 80 mL). The organic phase was dried over $MgSO_4$, the solvent removed, and the resulting a yellow oil was purified with column chromatography over silica with cyclohexane:dichloromethane (4:1) as eluent to give white crystals (2.628 g). ^{1}H-NMR ($CDCl_3$, 500 MHz, 25 °C) δ (ppm): 7.20 (s, 1H), 6.99 (s, 2H), 4.00 (q, 2H, ^3J = 6.9 Hz), 3.05 (s, 2H), 1.39 (t, 3H, ^3J = 6.9 Hz).

Diethyl 3,3′-((5-ethoxy-1,3-phenylene)bis(ethyne-2,1-diyl))dibenzoate (D). Ethoxy-3,5-diethynylbenzene (2.600 g, 15.3 mmol) was dissolved in triethylamine (60 mL) and purged with argon. Tetrakis(triphenylphosphine)palladium(0) (0.400 g, 0.35 mmol) and copper (I) iodide (0.140 g, 0.74 mmol) were added under argon flow. Ethyl 3-iodobenzoate (5.22 mL, 32.1 mmol) was added and the resulting pale yellow suspension was heated to 75 °C, and stirred overnight under argon. Triethylamine was removed with a rotary evaporator, and the slurry dissolved in CH_2Cl_2 (150 mL). This solution was washed with sat. NH_4Cl (3 × 100 mL) and H_2O (3 × 100 mL). The organic phase was dried over $MgSO_4$ and reduced to dryness on the rotary evaporator giving a yellow solid. This solid was washed with hexane, giving a beige powder. The beige powder was purified with column chromatography over silica with pure CH_2Cl_2 as eluent. The obtained cream coloured solid was dissolved in a minimum amount of CH_2Cl_2, and a large excess of cold MeOH added, causing precipitation of a white powder (3.874 g). ^{1}H-NMR ($CDCl_3$, 500 MHz, 25 °C) δ (ppm): 8.21 (s, 2H), 8.01 (d, 2H, ^3J = 7.7 Hz), 7.69 (d, 2H, ^3J = 7.6 Hz), 7.44 (t, 2H, ^3J = 7.6 Hz), 7.33 (s, 1H), 7.06 (s, 2H), 4.40 (q, 4H, ^3J = 7.1 Hz), 4.07 (q, 2H, ^3J = 6.6 Hz), 1.42 (m, 9H).

3,3′-((5-ethoxy-1,3-phenylene)bis(ethyne-2,1-diyl))dibenzoic acid (EtOLH₂). Diethyl 3,3′-((5-ethoxy-1,3-phenylene)bis(ethyne-2,1-diyl))dibenzoate (0.850 g, 1.82 mmol) was dissolved in THF/MeOH (2:1, 60 mL), and 10 mL of 2M aqueous solution of KOH was added. The mixture was stirred at 55°C for five hours. The organic solvents were removed with a rotary evaporator, and the remaining basic solution was acidified to pH = 1 with HCl (6 M), yielding a white suspension. This was filtered, and the white powder was washed with water (3×100 mL), and then dried under vacuum at 50°C. Yield 0.650 g, 87%. Elemental Analysis (%) Found (Calc.): C 75.94 (76.09), H 4.59 (4.42). ^{1}H-NMR (DMSO-d_6, 500 MHz, 25 °C) δ (ppm): 13.26 (br, 2H), 8.10 (s, 2H), 7.99 (d, 2H, ^3J = 7.7 Hz), 7.81 (d, 2H, ^3J = 7.6 Hz), 7.59 (t, 2H, ^3J = 7.7 Hz), 7.40 (s, 1H), 7.21 (s, 2H), 4.13 (q, 2H, ^3J = 6.7 Hz), 1.35 (t, 3H, ^3J = 6.8 Hz). ^{13}C-NMR (DMSO-d_6, 125 MHz, 25 °C) δ (ppm): 166.5, 158.6, 135.4, 132.1, 131.4, 129.6, 129.2, 126.7, 123.6, 122.3, 118.0, 89.0, 88.8, 63.7, 14.5.

ii) Complexes.

[$Cu_4(EtOL)_4(H_2O)_2(DMF)_2$] (1-DMF). A solution of $EtOLH_2$ (20 mg, 0.05 mmol) in DMF (1 mL) was added to a solution of $Cu(OAc)_2{\cdot}H_2O$ (9.6 mg, 0.05 mmol) in DMF (1 mL). The resulting blue solution was left to stand at room temperature. Blue block crystals suitable for single crystal X-ray diffraction formed after several hours. Yield 49.0 mg, 62%. A bulk sample

was also synthesized from DMA as follows: a solution of $EtOLH_2$ (200 mg, 0.50 mmol) in DMA (4 mL) was added to a solution of $Cu(OAc)_2 \cdot H_2O$ (97 mg, 0.5 mmol) in DMA (4 mL). The resulting blue solution was stirred at room temperature for 1 hour before MeOH (8 mL) was added. Over the course of several hours, a blue crystalline powder formed that was isolated by filtration, washed with DMA/MeOH (1:1, 2 × 5 mL), and dried in air, yielding 331 mg of a blue powder.

1-MeOH. 1-DMF or the bulk sample from DMA was transformed into the corresponding methanolic phase by soaking the samples in MeOH for seven days, replacing the MeOH each day with fresh solvent. Yield (starting from 49.0 mg of fresh **1-DMF**) 27.6 mg, 92%. Elemental Analysis for **1**$\cdot(MeOH)_2(H_2O)_2$ (%) Found (Calc.): C 64.31 (64.04), H 3.66 (3.85).

$[Cu_4(EtOL)_4(H_2O)_2(THF)_2]\cdot$10 THF (1-THF). The activated sample **1-a** was recrystallised from hot THF (approximately 4 mg/mL THF). Yield (starting from 6 mg of **1-a**) 3.9 mg, 34%. Upon cooling, blue block crystals suitable for single crystal X-ray diffraction formed.

$[Rh_4(EtOL)_4(H_2O)_2(DMA)_2][Rh_4(EtOL)_4(MeOH)_4]$ (2-DMA). $EtOLH_2$ (198 mg, 0.48 mmol), $[Rh_2(OAc)_4(MeOH)_2]$ (100 mg, 0.20 mmol), and Na_2CO_3 (53 mg, 0.50 mmol) were suspended in DMA (7 mL), sealed in a vial, and heated at 100 °C for 72 hours. After cooling to RT, the Na_2CO_3 was separated from the resulting green suspension by decantation, and the green suspension was centrifuged. Single crystals of **2-DMA** were obtained after one week by layering a 1 mL aliquot of the supernatant with MeOH (1:1, v/v). MeOH (30 mL) was added to the remaining supernatant, and the solid isolated by centrifugation. This green solid was washed with DMA, and then left to stand in MeOH for one week, replacing the MeOH each day, yielding the phase **2-MeOH** (55 mg, 26%, based on Rh). Elemental Analysis for **2**$\cdot(MeOH)_2(H_2O)_4$ (%) Found (Calc.): C 58.24 (58.36), H 3.47 (3.70).

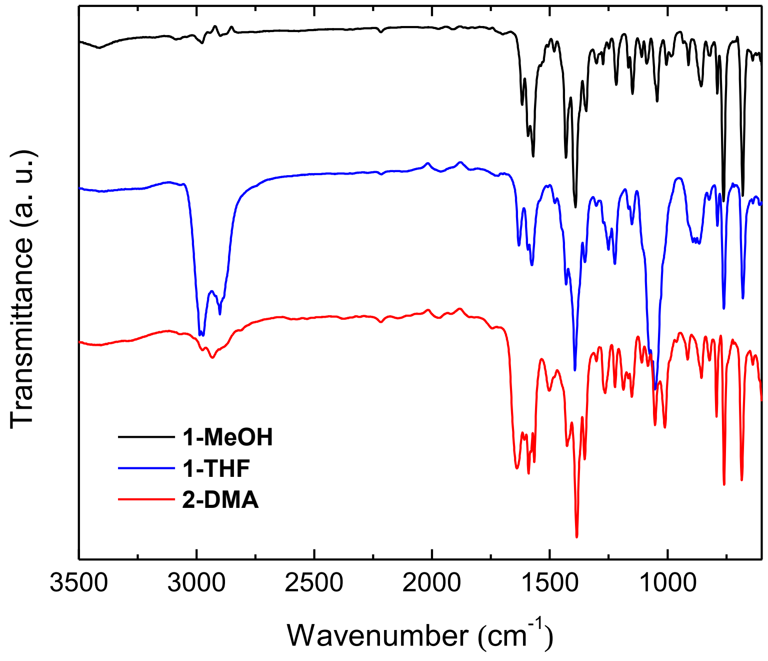

Fig. S1. IR-spectra for the compounds described in the text.

Physical characterization

^{1}H- and ^{13}C-NMR spectra were measured with a Bruker Ultrashield 500 plus (500 MHz) spectrometer at 25°C. Infra-red spectra were collected on neat samples using a Jasco FT/IR-6100 spectrometer. Thermogravimetric analyses (TGA) were performed with a Rigaku model Thermo plus EVO under an atmosphere of N_2, and using a heating rate of 5 °C min^{-1}. Powder X-ray diffraction data were collected at room temperature using a Rigaku SmartLab diffractometer equipped with Cu Kα radiation (λ = 1.54056 Å) – details of the *in situ* measurements are given below. Gas sorption isotherms were measured at 77 K (N_2) and 195 K (CO_2) using a BELSORP-max volumetric adsorption instrument from BEL Japan, Inc.. The samples were activated by heating for 16 hours under vacuum at a temperature of 120 °C.

In situ gas adsorption-PXRD

In situ gas adsorption−PXRD measurements for **1-a** and **2-a** were performed on a Rigaku SmartLab diffractometer with Cu Kα radiation connected to a BELSORP-18PLUS volumetric adsorption instrument (BEL Japan, Inc.). The instruments were automated and synchronised with each other, and an X-ray diffraction pattern was obtained at each equilibrium point in the adsorption isotherm.

Single crystal XRD

All crystallographic intensity data were collected using a Rigaku model XtaLAB P200 diffractometer equipped with a Dectris model PILATUS 200K detector and confocal monochromated Mo Kα radiation (λ = 0.71075 Å). All structures were solved with the ShelXT structure solution program using intrinsic phasing, and refined with ShelXL using least squares minimisation (Table S1).[S1] For all three structures, the SQUEEZE[S2] algorithm in PLATON[S3] was used to account for areas of diffuse solvent. For **1-DMF**, all non-hydrogen atoms were refined anisotropically. Hydrogen atoms were placed in calculated positions and refined with a riding model. Distance restraints were required for the DMF molecule coordinated to paddlewheel of the MOP, and also for both orientations of the aromatic ring disordered over two positions in a ratio of 0.55:0.45. The disordered solvent molecules in the lattice were accounted for using SQUEEZE. For **1-THF**, five molecules of THF were located in the lattice and included in the model, with remaining solvent accounted for with SQUEEZE. Hydrogen atoms were placed in calculated positions and refined with a riding model. All non-hydrogen atoms were refined anisotropically. Distance restraints were required for the disordered THF molecules. **2-DMA** was found to be highly disordered. All coordinated solvent molecules were refined isotropically, and their hydrogen atoms were omitted from the model. There are two distinct MOP molecules in the lattice: one with the formula $[Rh_4(EtOL)_4(MeOH)_4]$ and another with the formula $[Rh_4(EtOL)_4(H_2O)_2(DMA)_2]$. In the former, one aromatic ring was found to be disordered over two positions, which required distance restraints and also the thermal ellipsoids of counterpart atoms to be constrained with the command EADP. An ethoxyphenyl-ring of this molecule was also found to be disordered over two positions. For the second MOP molecule, one of the ethoxy- groups was very badly disordered, and only one orientation could be found with a partial occupancy fixed at 0.3 after trial refinements. This part of the molecule was refined isotropically, and the hydrogen atoms omitted. The remaining disordered solvent in the lattice was accounted for using SQUEEZE. For all crystallographic figures presented, only the major component of disordered moieties is shown. The .cif files for these structures have been submitted to the Cambridge Structural Database: CCDC 1840858-1840860. These

data can be obtained free of charge from The Cambridge Crystallographic Data Centre via www.ccdc.cam.ac.uk/data_request/cif.

Table 1. Selected crystallographic data for the crystal structures presented in the main text.

	**1-DMF**	**1-THF**	**2-DMA**
***λ* (Å)**		0.71073	
***T* (K)**	93	97	92
**Crystal System**	Triclinic	Monoclinic	Triclinic
**Space Group**	*P*−1	$P2_1/n$	*P*−1
***a* (Å)**	15.9653(3)	18.5382(2)	17.9146(2)
***b* (Å)**	17.6910(4)	25.3600(2)	18.0902(3)
***c* (Å)**	18.2258(5)	19.6509(2)	27.6891(3)
***α* (°)**	61.81(1)	90	77.35(1)
***β* (°)**	88.11(1)	116.08(1)	83.13(1)
***γ* (°)**	64.13(1)	90	61.95(1)
**V (Å^3)**	3983.8(2)	8297.8(1)	7725.4(2)
Z	1	2	2
**D_{calc} (g cm^{-3})**	0.863	1.116	0.935
**Reflections**	98887	85503	181555
**Unique Data**	14398	17542	25263
**R_{int}**	0.041	0.055	0.053
**$R[F^2 > 2\sigma(F^2)]$**	0.041	0.047	0.057
**$wR(F^2)$**	0.117	0.130	0.169
S	1.03	1.03	1.04
**ρ_{max}, ρ_{min} (eÅ^{-3})**	0.82,−0.33	0.93,−0.54	2.45,−1.14

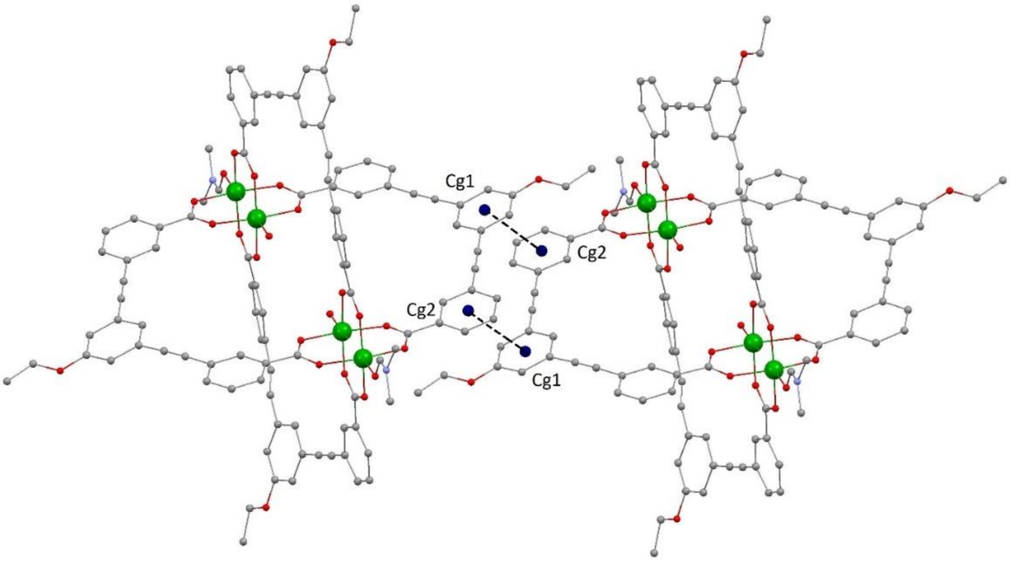

Figure S2. View of the shortest π···π stacking interaction formed in the lattice of **1-DMF**. The labels Cg1 and Cg2 refer to the centroids of the aromatic rings, as defined by the program PLATON (ref). The distance marked between the centroids measures 3.823(2) Å, while the nearest interatomic distance is 3.288(3) Å between C8 and C13.

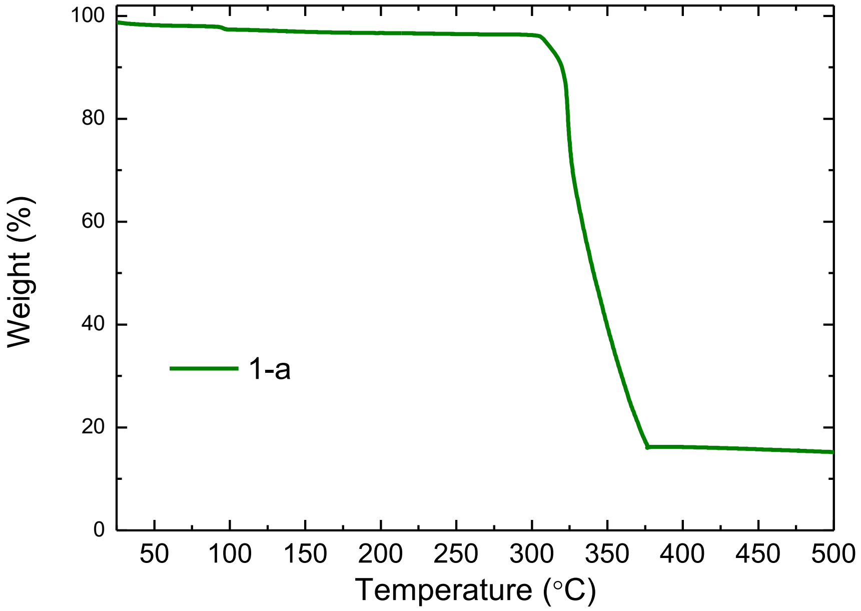

Figure S3. Thermogravimetric analysis (TGA) data for the activated phase **1-a**, showing that after activation of **1-MeOH** there is no solvent remaining in the lattice, with heating beyond 300 °C causing decomposition of the complex.

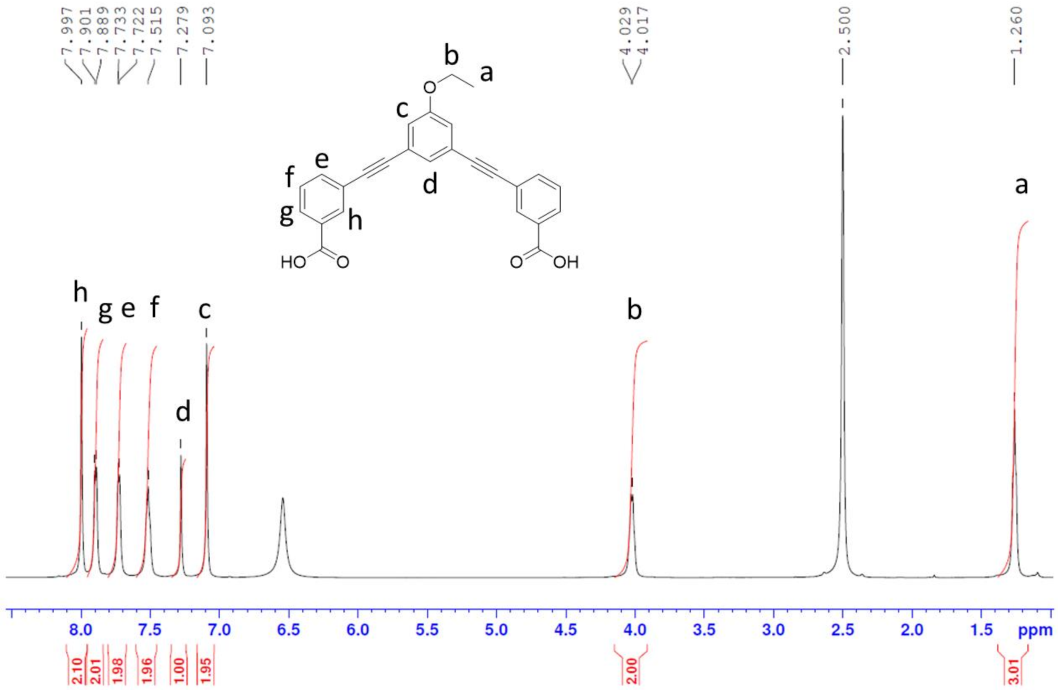

Figure S4. ^{1}H-NMR spectrum of the activated phase **1-a** subsequent to digestion in DCl/DMSO. The peak for C<u>H</u>3OH arising from residual MeOH would be expected at 3.28 ppm.

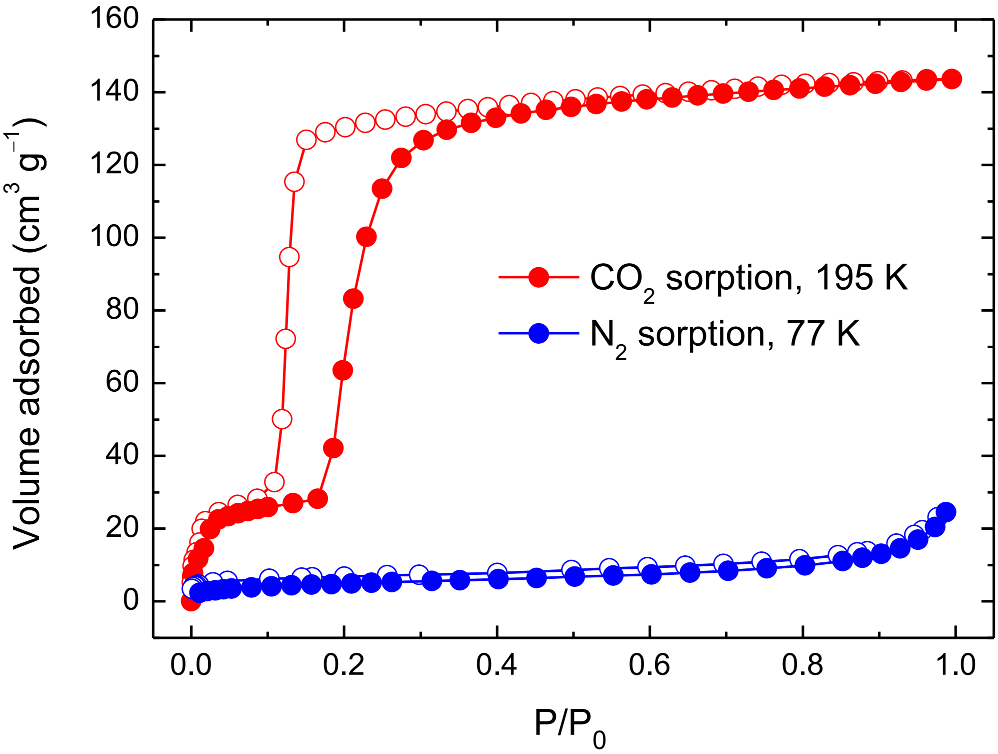

Figure S5. Gas adsorption isotherms for **1-a**. Filled symbols represent adsorption, while empty symbols represent desorption.

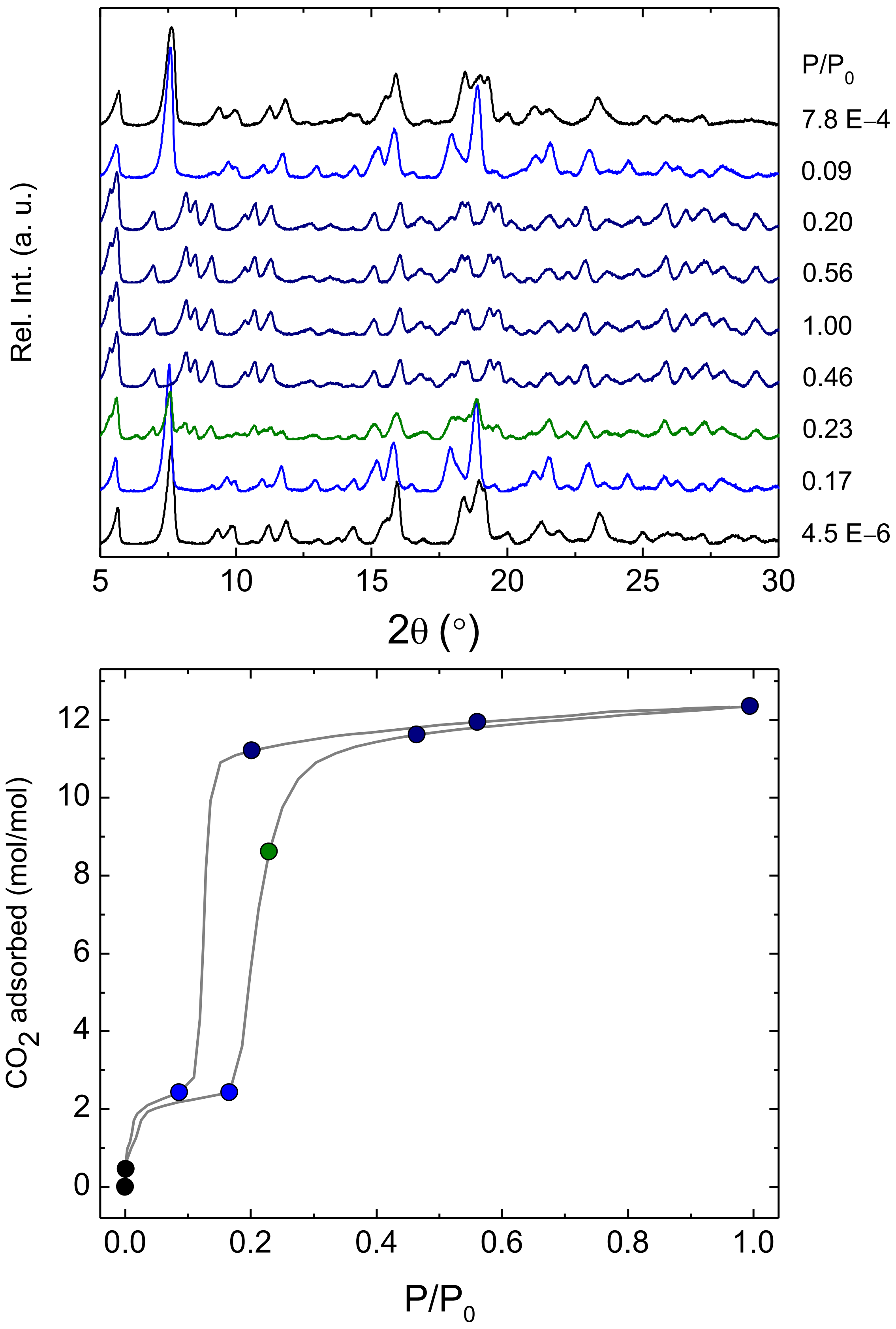

Figure S6. (top) *In situ* PXRD data collected during CO_2 gas sorption experiments at 195 K for **1-a**. The pressure at which each diffractogram was collected is indicated and highlighted on the accompanying isotherm (bottom).

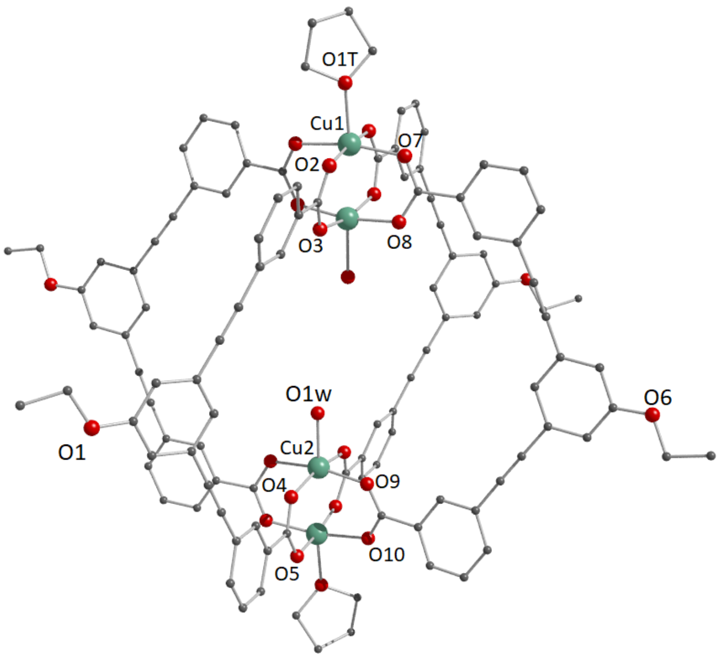

Figure S7. View of the molecular structure of the MOP molecule in **1-THF**. Hydrogen atoms have been omitted for clarity, and only crystallographically independent metal ions and heteroatoms have been labelled.

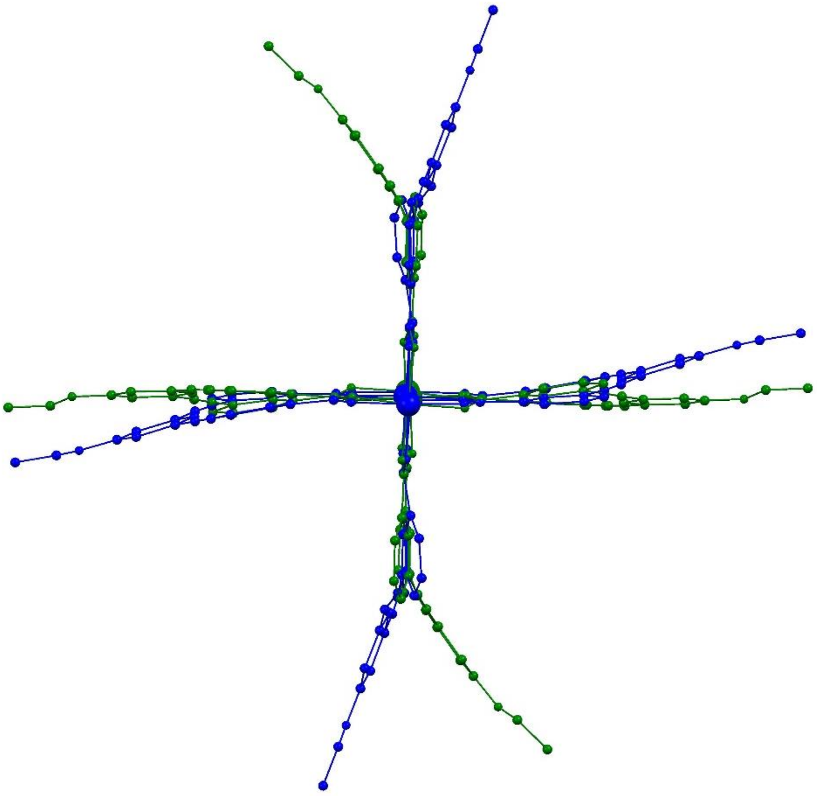

Figure S8. Overlay view of the MOP molecule **1** from the phases **1-DMF** (blue) and **1-THF** (green). Solvent molecules and hydrogen atoms have been omitted for clarity.

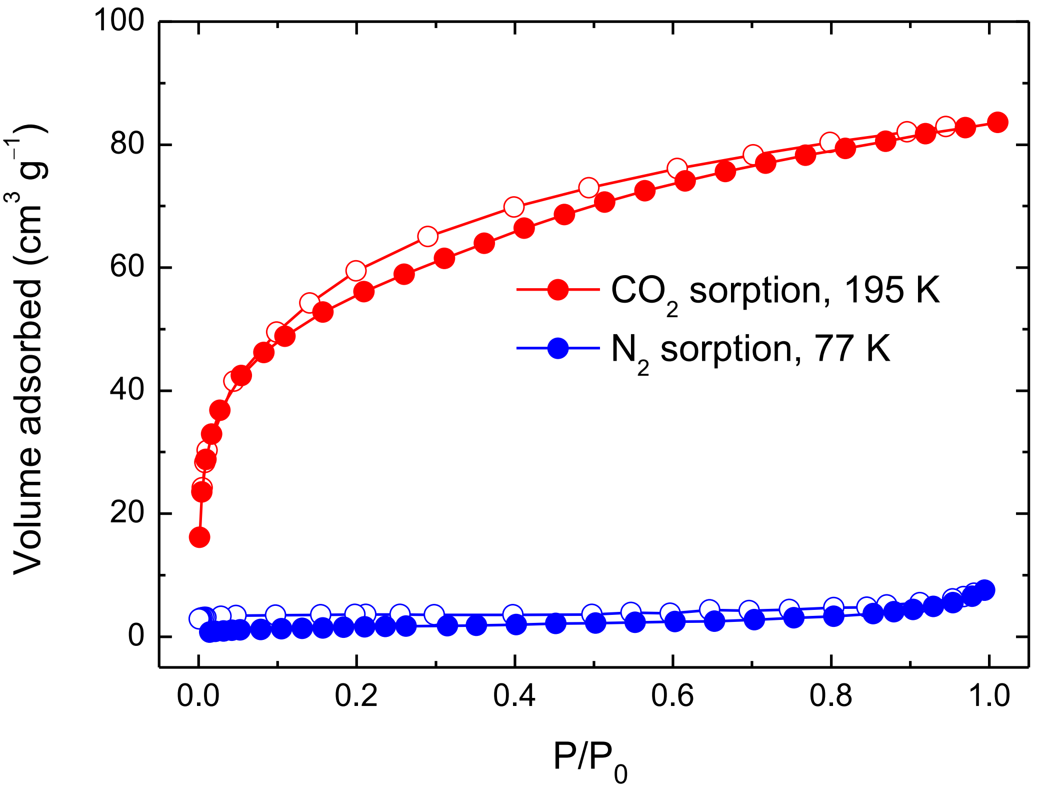

Figure S9. Gas adsorption isotherms for **1-b**. Filled symbols represent adsorption, while empty symbols represent desorption.

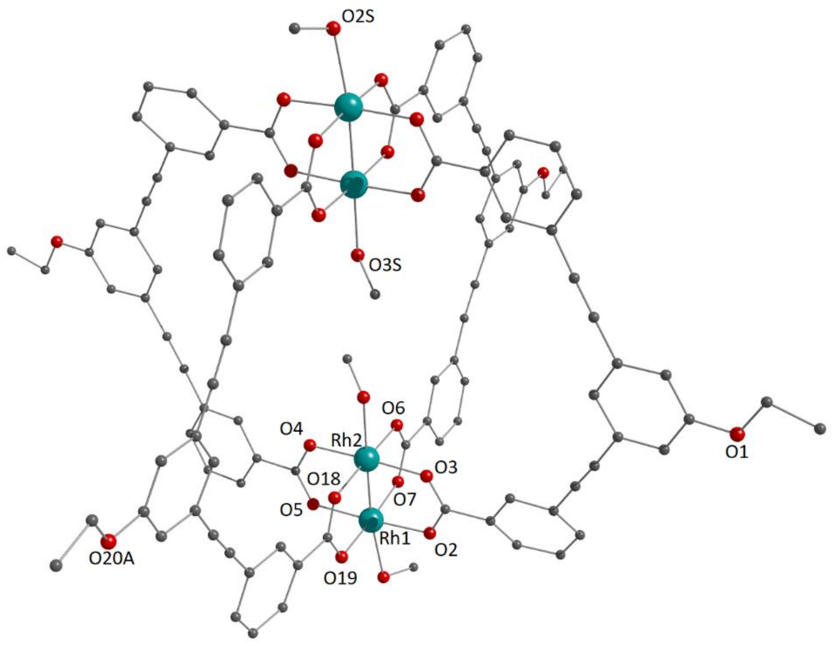

Figure S10. View of the molecular structure of one of the MOP molecules in **2-DMA**. Hydrogen atoms have been omitted for clarity, and only crystallographically independent metal ions and heteroatoms have been labelled.

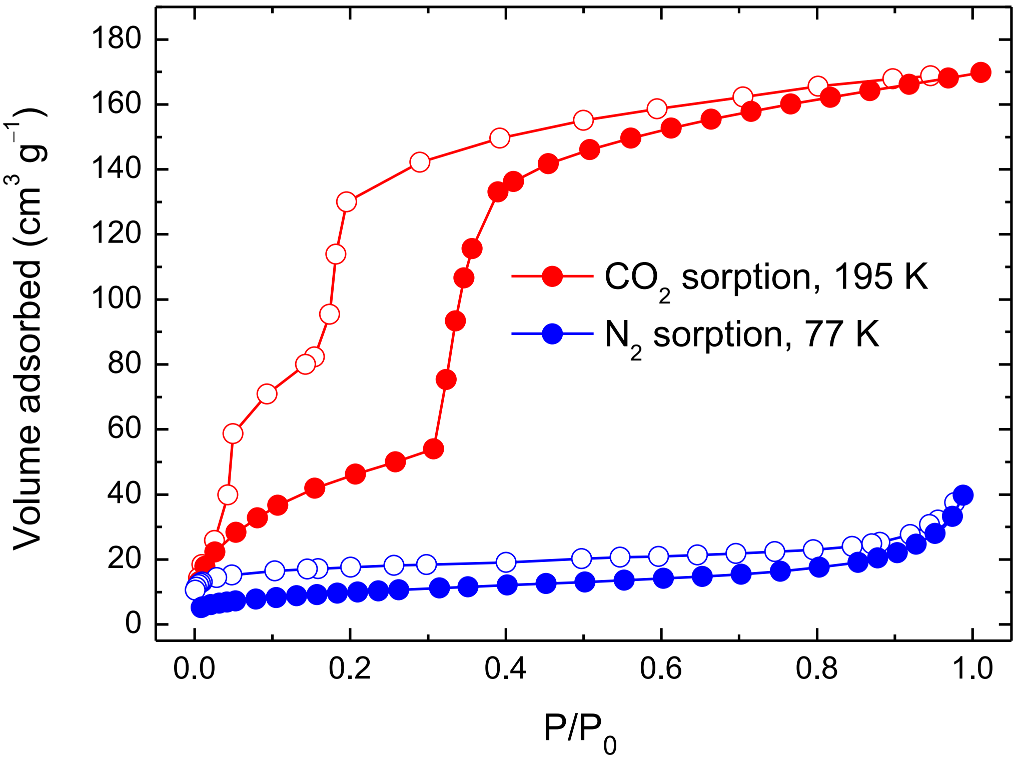

Figure S11. Gas adsorption isotherms for **2-a**. Filled symbols represent adsorption, while empty symbols represent desorption.

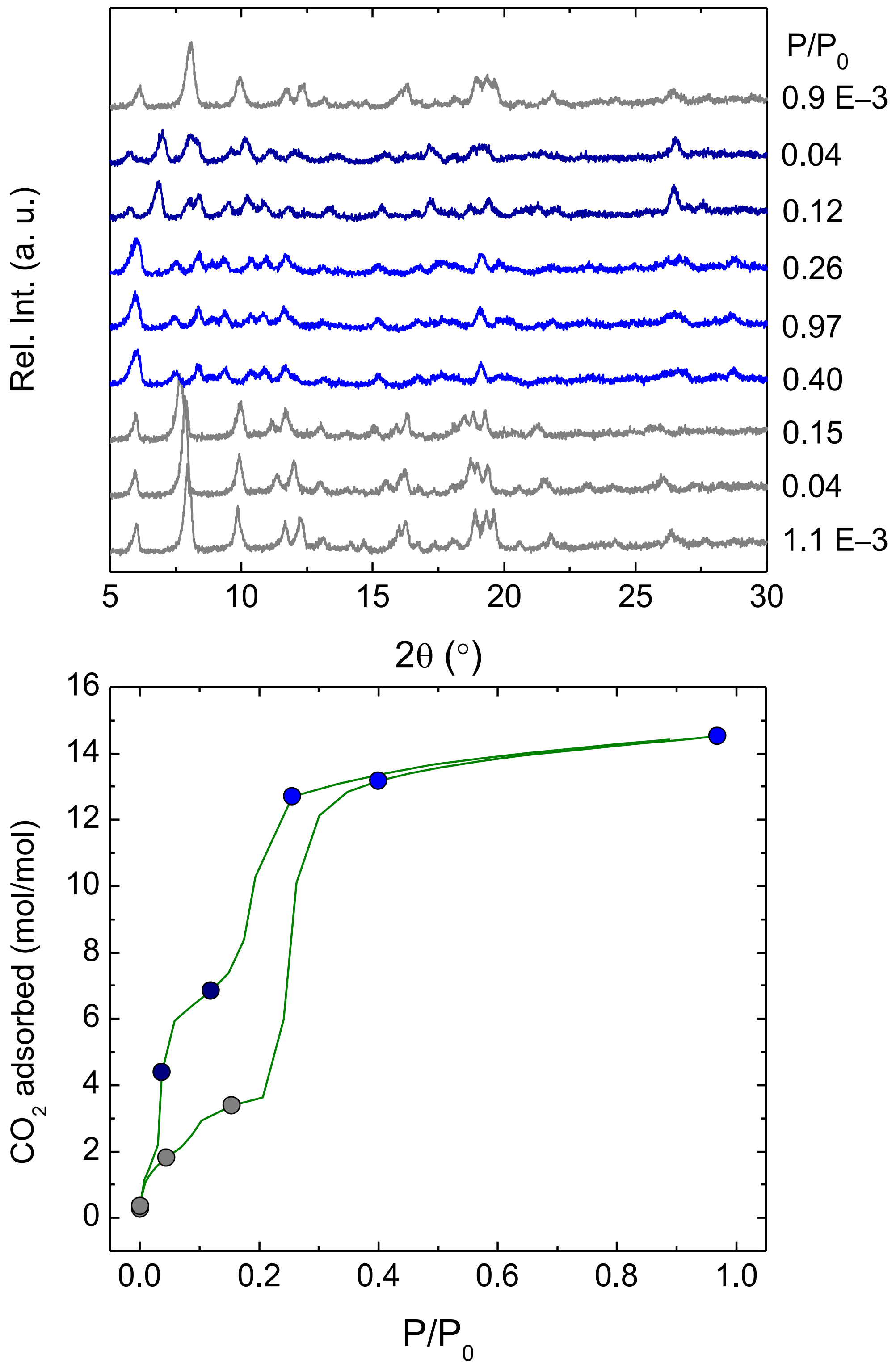

Figure S12. (top) *In situ* PXRD data collected during CO_2 gas sorption experiments at 195 K for **2-a**. The pressure at which each diffractogram was collected is indicated and highlighted on the accompanying isotherm (bottom).

References

S1. Sheldrick, G. M. (2015). *Acta Cryst.* A**71**, 3-8.

S2. Spek, A. L. (2015). *Acta Cryst.* C**71**, 9-18.

S3. Spek, A. L. (2009). *Acta Cryst.* D**65**, 148-155.
